# Outcomes of Open Fronto-Facial Resection for Fungal Osteomyelitis of Frontal Bone

**DOI:** 10.5041/RMMJ.10484

**Published:** 2022-10-27

**Authors:** Rupa Mehta, Karthik Nagaraga Rao, Nitin M. Nagarkar, Anil Sharma, Badal Kumar, P. Karthik

**Affiliations:** 1 Department of Otolaryngology and Head Neck Surgery, All India Institute of Medical Sciences, Raipur, India; 2 Department of Head and Neck Oncology, All India Institute of Medical Sciences, Raipur, India; 3 Director and CEO, All India Institute of Medical Sciences, Raipur, India; 4 Department of Neurosurgery, All India Institute of Medical Sciences, Raipur, India

**Keywords:** COVID-19, craniofacial resection, frontal sinus, fungal osteomyelitis, mucormycosis

## Abstract

**Introduction:**

The second wave of coronavirus disease 2019 (COVID-19) led to the resurgence of opportunistic infections due to the injudicious use of steroids. Sinonasal mucormycosis was declared an epidemic in India during the pandemic. Mucormycosis was managed effectively by surgical debridement along with systemic amphotericin B. Currently, a resurgence of mucormycosis following initial treatment, in the form of fungal osteomyelitis of the frontal bone, is being seen in India.

**Methods:**

This prospective study included 10 patients with fungal osteomyelitis of the frontal bone due to mucormycosis. All patients underwent surgical debridement of the sequestrum and involucrum, with systemic antifungal pharmacotherapy.

**Results:**

The average duration of time until mucormycosis recurrence was 22 days following initial treatment (range 10–33 days). Patients presented with extracranial bossing following outer frontal cortex erosion (*n*=3), bicortical erosion (*n*=3), bifrontal involvement (*n*=2), dural involvement (*n*=3), and involvement of the brain parenchyma and prefrontal cortex (*n*=2). All cases underwent debridement of the entire sequestrous bone and involucrum until normal bone could be identified. The mean admission duration was 4 weeks (range 3–6 weeks). All treated patients are currently alive and without disease, confirmed by contrast-enhanced computed tomography.

**Conclusion:**

Based on our experience, the successful treatment of fungal osteomyelitis due to mucormycosis requires a four-pronged approach: early detection, multidisciplinary management of comorbidities, surgical debridement of necrotic bone, and adequate systemic antifungal therapy.

## INTRODUCTION

In early 2021, severe acute respiratory syndrome coronavirus 2 (SARS-CoV-2) mutated and the world saw the emergence of the delta variant (B.1.617.1), which led to a deadly resurgence of the disease; the increased mortality was due to its high infectivity and virulence.^1^ Secondary infections or coinfections posed some critical challenges that increased the overall morbidity and mortality. Opportunistic infections such as mucormycosis were seen as focal outbreaks. Unfortunately, India has seen extremely high numbers of mucormycosis cases following SARS-CoV-2 infection, notably following the second wave of the pandemic (March 2021).^2^ Due to an exponential rise in the incidence of mucormycosis, the government of India declared it to be an epidemic during a pandemic.^3^

Mucormycosis was managed effectively by surgical debridement along with systemic amphotericin B through the multidisciplinary hospital teams across India.^4^ However, following the initial treatment of mucormycosis, a resurgence of the disease has been noted, in the form of fungal osteomyelitis of the frontal bone. This form of mucormycosis has not been previously recognized, and only a very few case reports have described it. The resurgence of this disease is attributed to incomplete clearance from previous surgery, inadequate amphotericin dosing, comorbidities, immune status, and previous disease extent.^4^,^5^

This paper presents the clinical features, radiological and intraoperative findings, and postoperative outcomes following the management of frontal osteomyelitis due to mucormycosis.

## SUBJECTS AND METHODS

This prospective, single-center, interventional, and cross-sectional study was conducted in the Department of Otorhinolaryngology and Head and Neck Surgery of the All India Institute of Medical Sciences in Raipur, India. Inclusion criteria were patients presenting with recurrent mucormycosis in the frontal bone following treatment for COVID-19-associated mucormycosis. Ten patients meeting those criteria were admitted and treated at our center between July 1, 2021 and August 30, 2021, and included in the study. Informed consent was obtained from all patients participating in the study. A detailed record of demographic data, clinical presentation, clinical investigations, points of pathogenesis, and pathogenesis management was acquired for each patient. Institutional ethical committee approval was obtained for the study (no. 1792/IEC-AIIMSRPR/2021, dated 19.07.2021). All procedures performed in the study were in accordance with the ethical standards given in the 1964 Helsinki Declaration and its later amendments; STROBE guidelines were utilized for data reporting.

The 10 patients underwent diagnostic nasal endoscopy to look for infection foci in the sinonasal cavity. Complementary cross-sectional radiological imaging was performed to determine disease extent and the degree of intracranial spread. The patients were scheduled for appropriate open surgical debridement based on disease extent. An intraoperative KOH fungal smear was performed to determine if mucormycosis was present. After ablative surgery, a definitive permanent plastic reconstruction procedure was deferred due to the possibility of disease recurrence and surgical field contamination. Patients diagnosed with mucormycosis were restarted on liposomal amphotericin B, irrespective of the previous cumulative dosing. High-risk patients were started on a prophylactic dose of low-molecular-weight heparin from postoperative day 1 to reduce thromboembolism incidence.^6^,^7^ The patients were started on isotonic nasal saline irrigation to clear the slough and debris upon nasal pack removal. Early recovery after surgery (ERAS) guidelines were strictly followed. All patients received pulmonary conditioning exercises, incentive spirometry, and chest physiotherapy. The patients were considered for discharge if they were clinically stable with reasonable control of comorbidities, had completed their target dosage of amphotericin B, and exhibited no radiological evidence of disease.

## RESULTS

A total of 10 patients (9 males, 1 female) were admitted for recurrent frontal osteomyelitis following initial treatment of COVID-19-associated sinonasal mucormycosis. A summary of the patient data, comorbidities, and disease extent and duration is provided in Table 1. Eight patients were diagnosed with recurrent mucormycosis after KOH mount of pus from the frontal bone osteomyelitis obtained intraoperatively (fungal elements/broad aseptate hyphae). All patients had been previously diagnosed with sinonasal mucormycosis and a previous history of SARS-CoV-2 infection; 3 patients had undergone previous orbital exenteration for COVID-19-associated mucormycosis (CAM).

**Table 1:** Case Descriptions of Mucormycosis Patients.

Age	Sex	Comorbidities	Mucormycosis Extent	Time to Recurrence after Initial CAM Therapy (days)	Extent of Present Disease	Intraoperative CSF Leak
62	M	DM + HTN	SNP	29	Left frontal sinus, outer frontal sinus table erosion, dura free, brain free	No
52	M	DM + HTN + hypothyroidism	SNOC	10	Bilateral frontal sinus, erosion of both frontal sinus tables, dural enhancement, prefrontal cortex involved	Yes
30	M	DM	SNO	19	Left frontal sinus, outer frontal sinus table erosion, dura free, brain free	No
43	M	DM	SNO	16	Bilateral frontal sinus, erosion of both frontal sinus tables, dural enhancement, prefrontal cortex involved	Yes
33	M	DM	SNC	26	Left frontal sinus, inner frontal sinus table erosion, dura free, brain free	No
40	F	DM + HTN	SN	26	Left frontal sinus, outer frontal sinus table erosion, dura free, brain free	No
45	M	DM + HTN	SN	31	Left frontal sinus, outer frontal sinus table erosion, dura free, brain free	No
43	M	DM	SNP	33	Left frontal sinus, inner frontal sinus table erosion, dura free, brain free	No
46	M	DM	SN	14	Left frontal sinus, outer frontal sinus table erosion, dura free, brain free	No
39	M	DM + HTN + hypothyroidism	SN	16	Left frontal sinus, outer frontal sinus table erosion, dura free, brain free	No

CAM, COVID-19-acquired mucormycosis; CSF, cerebrospinal fluid; DM, diabetes mellitus; F, female; HTN, hypertension; M, male; SN, sinonasal; SNC, sino-naso-cerebral; SNO, sino-naso-orbital; SNOC, sino-naso-orbito-cerebral; SNP, sino-naso-palatal.

**Figure 1:**
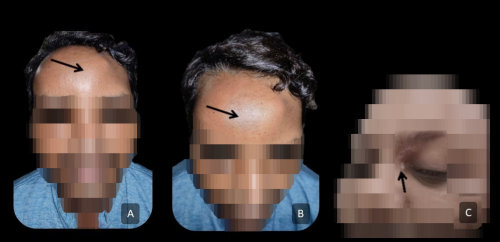
Physical Characteristics of Patients with Frontal Bossing (A and B) and Discharging Sinus (C). **A and B:** Frontal bossing, which is similar to Pott’s puffy tumor. **C:** The discharging sinus is adjacent to the medial canthal region.

The average duration of time until mucormycosis recurrence was 22 days following the initial treatment (range, 10–33 days). The patients presented with frontal headache (*n*=10), frontal bulge (*n*=6), discharging sinus near the medial canthus (*n*=1; Figure 1), and fever (*n*=4). Diagnostic nasal endoscopy commonly revealed mucosalization of the nasal cavity with mild polypoidal changes in the opened sinuses; there was evidence of pus discharge from the frontal ostium in 3 cases, and 4 cases had frontal outflow tract obstruction. Retrospective radiographic review of these patients revealed partial to complete opacification of the frontal sinus in 3 cases during initial recurrent disease presentation.

**Figure 2:**
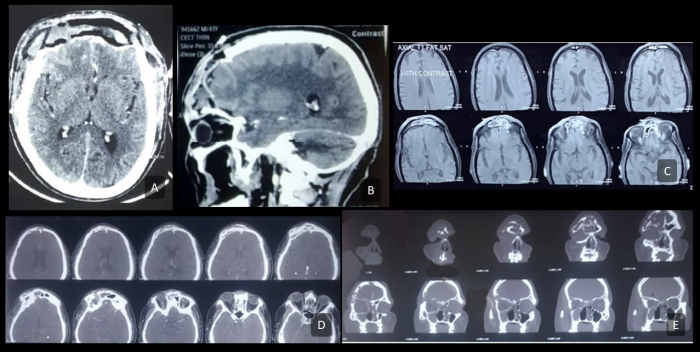
Pre-operative Imaging. **A and B:** Axial and sagittal contrast-enhanced CT, respectively, showing complete erosion of frontal bone with necrotic soft tissue debris between the skin and dura; the dura appears enhanced. **C:** T2-weighted axial MRI cuts depicting heterogeneously enhancing soft tissue density in the frontal sinus with posterior table erosion. **D and E:** Axial and coronal contrast-enhanced CT cuts, respectively, depicting the erosion of anterior and posterior tables of frontal sinus, suggestive of osteomyelitis.

Contrast-enhanced computed tomography was performed to look at the bony status, and complementary magnetic resonance imaging was used as a problem-solving tool to determine soft tissue extent (intraorbital, orbital apex, and intracranial extension) and the presence of intra- or extradural intracranial extension. Cross-sectional imaging revealed extracranial bossing following outer frontal cortex erosion in 3 cases (Figure 2), bicortical erosion in 3 cases, bifrontal involvement in 2 cases, dural involvement in 3 cases, and brain parenchymal and prefrontal cortex involvement in 2 cases (Table 1).

**Figure 3:**
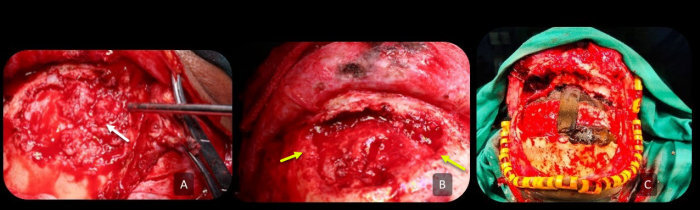
Intraoperative Photographs Depicting the Fungal Debris. **A:** Sequestrum (white arrow) and involucrum in the frontal sinus posterior table. **B:** Debridement of necrotic tissue until the normal bone is reached (yellow arrows). **C:** Iodoform ribbon gauze packed in the frontal sinus following extensive debridement; the dura is intact.

Open surgical debridement was preferred due to the recurrent nature of the disease and for adequate disease clearance. Bifrontal craniotomy with complete debridement of the frontal bone and pericranial flap was performed in 7 patients and an ipsilateral supraorbital craniotomy in 2 patients; intraoperative cerebrospinal fluid (CSF) leak was seen in 2 patients and was repaired by primary closure with onlay of the pericranial flap. Free flaps and alloplastic materials were not used for reconstruction due to the possibility of seeding the graft, leading to a foreign body reaction. Frank pus was seen in all patients after exteriorizing the frontal sinus. Bony sequestrum and involucrum were also seen along the frontal bone in all cases (Figure 3). Debridement entailed removal of entire sequestrous bone and a part of the involucrum until normal bone could be identified.

None of the patients experienced postoperative CSF leak. A closed suction drain was placed in the cavity and then removed if the amount of drainage was less than 25 mL. Nasal tamponade packing was removed 48 hours postoperatively.

**Figure 4:**
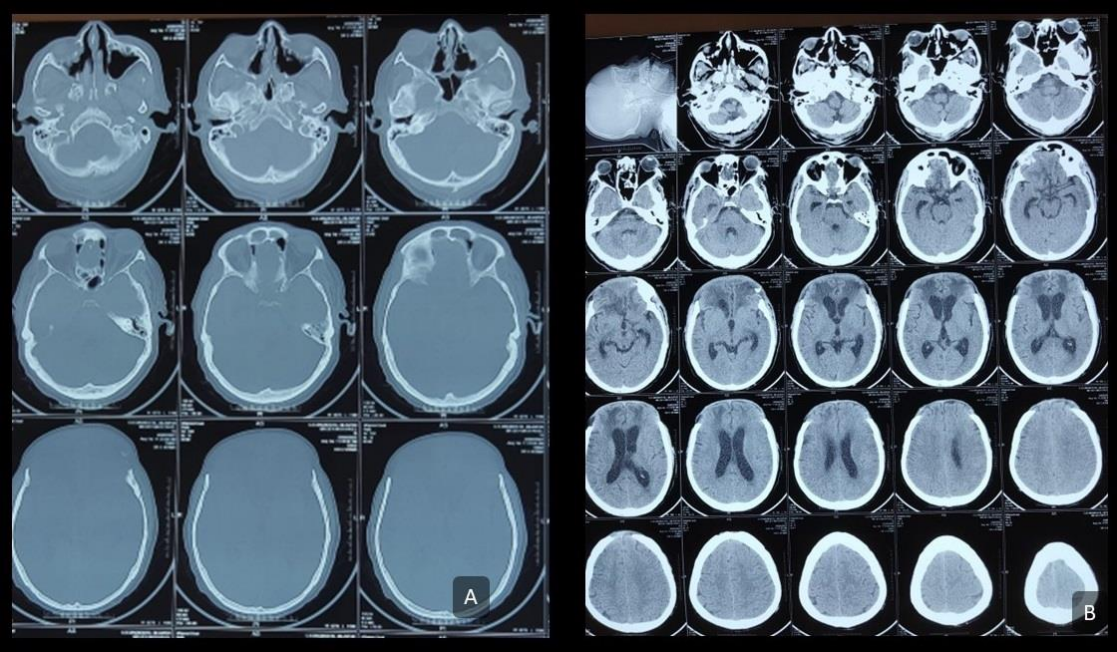
Computed Tomography Scan at 9 Months After Surgery. **A:** Debrided frontal bone in axial bony window cuts. **B:** No abnormal soft tissue density is found in the operative site, suggesting locoregional disease control.

**Figure 5:**
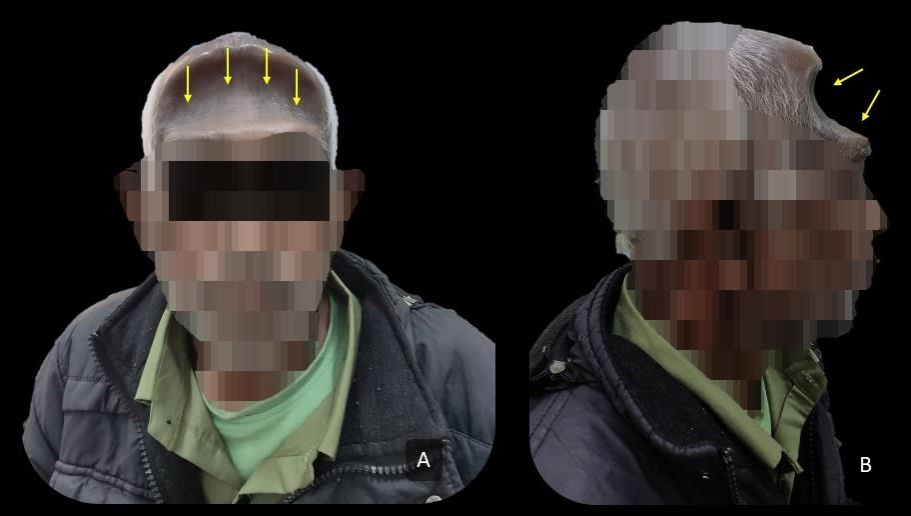
Follow-up Photograph of the Patient Showing Significant Frontal Hollow Due to Extensive Debridement. The patient has no residual neurological deficits.

None of the patients experienced neurological deficits following the surgery; 2 patients had local wound collection that required exploration and evacuation under anesthesia (Clavien–Dindo grade 3b). All the patients received prophylactic broad-spectrum antibiotic coverage with ceftriaxone and metronidazole for 7 days, or until culture sensitivity reports were negative, together with antifungal treatment with amphotericin B. The prophylactic antibiotics were given to prevent infection at the surgical site as per the institutional rational antibiotics usage policy; the surgical wounds were Centers for Disease Control and Prevention (CDC) class III contaminated.^8^ Amphotericin B was continued until radiological disease clearance was seen. The mean duration of admission was 4 weeks (range, 3–6 weeks). At 9 months’ follow-up, all 10 patients were alive and disease-free, confirmed by cross-sectional imaging (Figure 4). Four of the patients have a significant frontal hollow due to the extensive debridement (Figure 5).

## DISCUSSION

Only a handful of case reports have described frontal sinus osteomyelitis following mucormycosis.^9^–^12^ Mucormycosis usually affects the nose and paranasal sinuses; the disease can spread via direct extension or by hematogenous spread. Hosseini and Borghei^13^ initially described the routes for spread of mucormycosis. We hypothesize the following pathways for the spread of mucormycosis into the frontal sinus:

1. Via the ethmoidal air cells by direct extension, leading to frontal outflow tract obstruction.
2. By erosion of the frontal sinus floor from superior part of the orbit.
3. Perineural spread along the supraorbital or supratrochlear nerves.
4. Along the anterior ethmoidal arteries.
5. Direct spread to the brain through the roof of the ethmoid and cribriform plate to the skull base/frontal bone to inferior aspect of the frontal bone.
6. Disease tracking along the orbital apex.

In acute osteomyelitis, the patient is toxic with tender swelling over the bone involved, called Pott’s puffy tumor. Pott’s puffy tumor was initially described as a complication of acute bacterial frontal sinusitis. The successful management of frontal bone osteomyelitis entails removal of the entire sequestrum and debriding the involucrum until the normal bony architecture is reached. Frontal sinus obliteration with the fat, muscle, and alloplastic materials has been tried with varying success. In the setting of mucormycosis, it is of utmost importance to clear the disease in its entirety, until fresh bleeding is observed. The main crux of frontal sinus cranialization or obliteration in the mucormycosis setting is to ensure complete disease elimination and dead space, which forms a protective barrier for the intracranial spread, enabling clinicoradiological reassessment of disease recurrence. The use of alloplastic materials is usually not recommended in the setting of severe infection.^14^

Frontal disease can be approached endoscopically with complete bony debridement, provided the disease is limited and diagnosed early.^15^ Treatment with amphotericin alone is not sufficient for local control. Thorough debridement must be performed until a healthy tissue margin is found; this may have to be repeated at regular intervals. Endonasal disease may have to be treated with comprehensive surgery such as wide resection of necrotic soft tissue and bone and exenteration of the orbit.^16^,^17^ According to the available literature, patients with intracranial extension are less likely to respond to radical surgery, and they have a poor prognosis.^18^ However, if surgery is performed early on, the chances of survival increase significantly.^15^ It has been well established that successful management of mucormycosis is dependent upon the management of comorbid conditions—this is the single most crucial factor determining overall patient prognosis.^19^

A study on the AmBiLoad trial noted that higher amphotericin B doses did not improve the overall outcome compared to conventional dosing.^20^ Before administering amphotericin B, factors such as existing diabetic and hypertension-induced nephropathy must be kept in mind. There is no strict consensus on the duration and the target dosage of amphotericin B therapy,^21^ especially in the recurrent setting. In this study, on the recommendations of our expert institutional multidisciplinary team, we titrated the target dose based on disease extent, comorbidities, and response to therapy.

In a recent systematic review, an 80% mortality was seen with central nervous system (CNS) involvement.^22^ Among the patients who have survived, debridement was performed for minimal or early invasion to the dura and brain. The overall better outcomes in our study can be attributed to early disease detection, prompt surgical debridement, prolonged amphotericin B administration, and strict management of comorbidities.

The main limitation of this study was the sample size; clearly, a larger number of patients would have improved the power of the study. However, a study that includes large numbers of patients may not be possible due to the rarity of recurrent mucormycosis of the frontal bone.

## CONCLUSION

In our study, we attribute the successful treatment of fungal osteomyelitis due to mucormycosis to a four-pronged approach: (1) early disease detection; (2) multidisciplinary management of comorbidities; (3) surgical debridement of necrotic bone; and (4) adequate systemic antifungal therapy. Long-term outcomes of fungal osteomyelitis of the frontal bone have not yet been established due to the relative rarity of this condition. Furthermore, any incomplete surgical clearance may lead to further recurrences. Hence, to achieve complete resolution of the condition, we believe that patients must be closely followed at regular intervals for at least two years.

## Abbreviations

COVID-19: coronavirus disease 2019.
